# Adult reference intervals for IgG subclasses with Siemens immunonephelometric assays in Chinese population

**DOI:** 10.1186/s13223-017-0216-7

**Published:** 2017-10-05

**Authors:** Ping Li, Zhongjuan Liu, Ziyan Wu, Xiaoting Wen, Liubing Li, Shulan Zhang, Yingchun Xu, Yongzhe Li

**Affiliations:** 1 Department of Rheumatology and Clinical Immunology, Peking Union Medical College Hospital, Chinese Academy of Medical Sciences & Peking Union Medical College, Key Laboratory of Rheumatology and Clinical Immunology, Ministry of Education, Beijing, 100730 China; 2 Department of Clinical Laboratory, Peking Union Medical College Hospital, Chinese Academy of Medical Sciences, Beijing, 100730 China

**Keywords:** Immunoglobulin subclasses, Immunonephelometric assays, Chinese population

## Abstract

**Objective:**

To determine the adult reference intervals for the Siemens IgG subclass reagents.

**Methods:**

636 blood samples of healthy adults were analyzed to determine the level of IgG subclass using the reagents of Siemens immunonephelometric assay with molecular biology kits.

**Results:**

IgGSc reference intervals were as follows: IgG1 4.45–9.76 g/L, IgG2 2.07–8.57 g/L, IgG3 0.08–0.80 g/L and IgG4 0.05–1.54 g/L. There was an excellent correlation between the total IgG and the sum of the IgG subclasses. No significant gender and age differences were observed.

**Conclusions:**

Our data provide the missing reference intervals and enable the use of the nephelometric IgG subclass reagents in Chinese. The study can offer reference on clinic diagnose.

## Background

Four distinct heavy chain subgroups of human IgG were first demonstrated in the 1960s by using polyclonal antisera prepared in animals immunized with human myeloma proteins [1, 2]. Since then, determination of selective immunoglobulin subclasses (IgGSc) has become an established tool in the diagnosis of many diseases, such as immunologic deficiencies and adult IgG4-related disease (IgG4-RD) [3, 4].

Recurrent respiratory tract infections that are poorly responsive to antibiotics are common presentations of immunoglobulin (Ig) deficiency in adults and children. There is a group of patients with similar clinical presentation, but who have serum IgG levels within or close to the bottom of the normal range and in whom one or more of the IgG subclasses 1–4 is entirely lacking or present in greatly reduced amounts [5, 6]. IgG4-RD is a recently recognized fibroinflammatory condition that has tumefactive lesions, a dense lymphoplasmacytic infiltrate rich in IgG4-positive plasma cells, and this disease is typically associated to an increase of serum IgG4 level. Serum IgG4 elevation becomes from this date a biological marker of IgG4-RD, and serum IgG4 elevation (> 1.35 g/L) is considered as a diagnosis criteria for IgG4-RD [7, 8].

The immunonephelometric determination of IgG subclasses in serum is based upon the specific reaction of the respective human IgG subclass with a polyclonal (mono) specific, highly avid anti-IgG subclass antiserum. Immunonephelometric assays were designed for fast, fully automated and specific quantification of human IgG subclasses profile (IgG1, IgG2, IgG3 and IgG4).

Although more and more serum IgG subclasses assays were performed by the Dade Behring IgG subclass reagents (Siemens Healthcare Diagnostics Products GmbH BN™) in China, a limitation of IgGSc is the lack of larger cohorts providing populational serum concentration measurements in order to establish normal reference values, and published reference intervals are limited to a certain geographical area. Therefore, the purpose of this study was to assess IgGSc reference intervals in healthy, mostly Chinese adults, using fully automated and reproducible immunonephelometric assays. We performed the whole experiment process according to the test method recommended by Clinical and Laboratory Standards Institute (CLSI) [9].

## Methods

### Information of the recruited subjects

The study population included 636 clinically healthy adults (309 males and 327 females) from 18 to 85 years of age who were admitted to Peking Union Medical College Hospital for health physical examination. Good health was confirmed by a clinical questionnaire and blood parameters within reference intervals. The serum was frozen within less than 4 h in sterile at −80 °C until measurement. All specimens included for analysis were the first received on each patient during the period, duplicates being excluded. The study was approved by the ethics committee of the Peking Union Medical College hospital and all subjects gave informed consent.

### Measurements of reference intervals

All IgGSc were measured by nephelometry according to the manufacturer’s instructions (BN2 nephelometer, Dade Behring GmbH, Marburg, Germany). The inter-assay coefficients of variation (CV) are 1.9–5.3% with total CV of 2.6–6.2%. We used the following concentrations for the CVs: Level SL-H: IgG1 = 7.33 g/L, IgG2 = 3.87 g/L, IgG3 = 0.482 g/L, IgG4 = 0.779 g/L; for Level SL-L: IgG1 = 3.78 g/L, IgG2 = 1.94 g/L, IgG3 = 0.191 g/L, IgG4 = 0.479 g/L; and for Level SL-M: IgG1 = 5.83 g/L, IgG2 = 2.85 g/L, IgG3 = 0.333 g/L, IgG4 = 0.686 g/L. Reagents used for these measurements were from the Siemens Healthcare Diagnostic Products GmbH BN (tm). IgG subclass assays were performed with N antiserum to human IgG, N AS IgG1, product code OQXI (lot no. 090167), N AS IgG2, product code OQXK (lot no. 090274), N Latex IgG3, product code OPAV (lot no. 086169), and N Latex IgG4, product code OPAU (086070), and the N protein standard SL (OQIM), which is based on the Sanquin (Amsterdam, Netherlands) nephelometric standard M1590. The sum of IgG subclasses 1–4 corresponds to the total IgG based on the certified European reference material ERM^®^-DA470.

### Statistical analysis

For statistical analysis patients were divided into three age groups: 18–45 years, 45–65 years and more than 65 years. We carried out comparisons between groups using Wilcoxon–Mann–Whitney test to determine whether the medians were dependent on age. Data were also examined within each age band for differences between male and female reference ranges. Statistical significance was assumed if p < 0.05. According to CLSI, outlying values were removed using Dixon’s Outlier Statistic [9]. ‘High’ and ‘low’ populations were excluded, leaving a ‘normal range’ (the reference population). To reflect the correlation between the sum of the four subclasses and total IgG through the Pearson correlation coefficient. Correlation analysis and graphics were created using Graph Pad Prism for Windows version 5.0 (Graph Pad Software, San Diego, CA, USA). Descriptive statistics and percentiles were calculated using SPSS software (version 17.0). Ranges for each variable were derived as the interval from the 2.5 to 97.5th centile of the sample measurements.

## Results

**Fig. 1:**
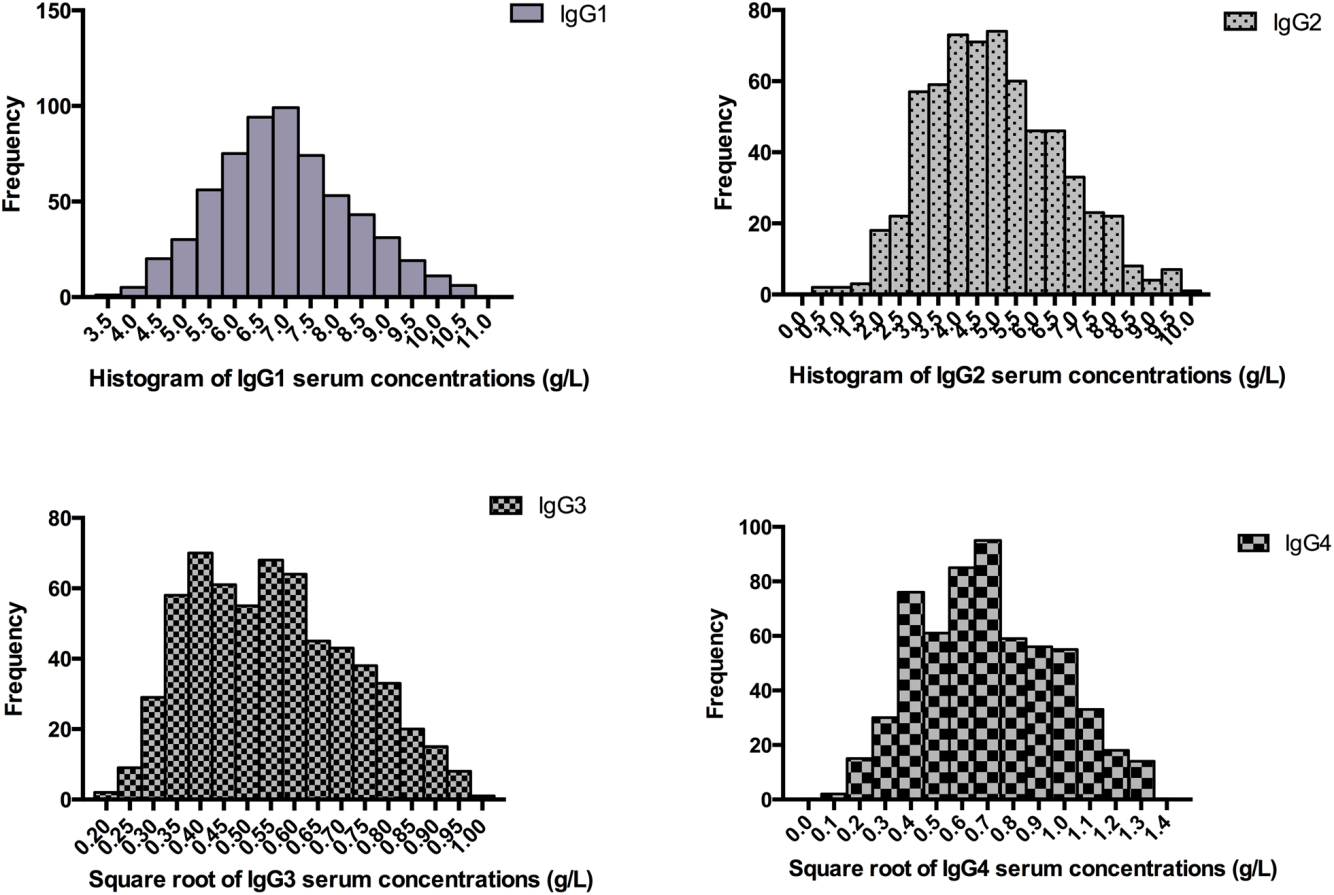
Histogram of square-root transformed IgG subclasses serum concentrations in 636 healthy adults

After excluding outliers, IgGSc serum concentrations were determined in 617 IgG1, 631 IgG2, 619 IgG3 and 599 IgG4 groups. IgG1 and IgG2 serum concentrations were normally distributed, and IgG3 and IgG4 serum concentrations were non-normally distributed, whereas square-root transformed serum concentrations were approximately normally distributed (Fig. 1).

Serum IgG1 ranged from 4.45 to 9.76 g/L with a median of 6.85 g/L, IgG2 ranged from 2.07 to 8.57 g/L with a median of 4.81 g/L, IgG3 ranged from 0.08 to 0.80 g/L with a median of 0.29 g/L, and IgG4 reference ranged from 0.05 to 1.54 g/L with a median of 0.46 g/L. The results can be seen in Table 1.

**Table 1:** The reference intervals for IgG subclasses

Groups	Median (g/L)	2.5th percentile (g/L)	25th percentile (g/L)	75th percentile (g/L)	97.5th percentile (g/L)	Reference interval
IgG1	6.85	4.45	6.04	7.83	9.76	4.45–9.76
IgG2	4.81	2.07	3.69	6.10	8.57	2.07–8.57
IgG3	0.29	0.08	0.17	0.46	0.80	0.08–0.80
IgG4	0.46	0.05	0.25	0.80	1.54	0.05–1.54

**Fig. 2:**
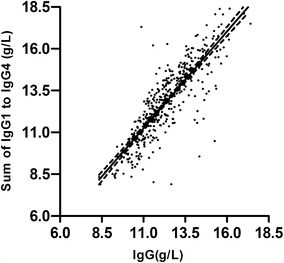
The relationship between total IgG and the sum of the IgG subclasses

The data showed no significant sex-related and age differences. We observed good correlation between the sum of the four subclasses and total IgG (r^2^ = 0.8527, p < 0.0001; Fig. 2).

## Discussion

Due to the fact that appropriate reference intervals are important for clinical application of diagnostic markers, it was necessary to produce reliable reference intervals for the newly developed commercial assays, especially as nephelometric data are scarce in Chinese populations.

Several studies have reported IgGSc concentrations in healthy adults. Most of the studies used RIDs and various reference materials [10, 11]. In this study, we used automated nephelometry to measure IgGSc values in healthy adults. The results were different from those reported for previous studies with the concentrations of IgG3 being greater that those of IgG4 [3]. Our data showed that the median of IgG3 was lower than the median of IgG4. The good correlation between the sum of the IgGSc and the measured value of total IgG supports the accuracy of the determinations.

Our results are different from the other reference ranges, some reasons could exist for this disparity. First reason may be the population difference, ethnicity has been shown to exert a significant role in both disease susceptibility and disease expression. Understanding population differences will improve our understanding of the laboratory indicators and improve clinical diagnosis. Second, most of the studies used RIDs and various reference materials, in our study, we used automated nephelometry to measure IgGSc values. Another reason is that we recruited 636 healthy adults, the number of sample size is more than the number of previous study. These factors may have led to the difference between our results and other reports.

According to International Federation of Clinical Chemistry (IFCC), it is necessary for every laboratory to have its own set of reference limits. Moreover, the reference values of this study were consistent with our previous study [12]. Our retrospective study clearly confirms that serum IgG4 elevation above 1350 mg/L is not specific for the diagnosis of IgG4-RD and can be observed in several clinical situations. Because of the large number of non-IgG4-RD diagnoses that were found to be associated with elevated IgG4 concentrations, we defined the optimal cut-off (1575 mg/L) by ROC curve for Chinese population, when all the IgG4-RD group and other diseases groups and healthy controls were compared. A cut-off level of 1575 mg/L can increase the specificity to 88.2% for distinguishing other diseases.

In summary, our data provide the missing Chinese adults reference intervals and enable the use of the Siemens nephelometric IgG subclass reagents in adults. With the current manuscript, we established adult reference intervals for Chinese Populations.
